# Method for 3D atomic structure determination of multi-element nanoparticles with graphene liquid-cell TEM

**DOI:** 10.1038/s41598-023-28492-5

**Published:** 2023-02-01

**Authors:** Junyoung Heo, Dongjun Kim, Hyesung Choi, Sungin Kim, Hoje Chun, Cyril F. Reboul, Cong T. S. Van, Dominika Elmlund, Soonmi Choi, Kihyun Kim, Younggil Park, Hans Elmlund, Byungchan Han, Jungwon Park

**Affiliations:** 1 School of Chemical and Biological Engineering and Institute of Chemical Processes, Seoul National University, Seoul, 08826 Republic of Korea; 2 Center for Nanoparticle Research, Institute for Basic Science (IBS), Seoul, 08826 Republic of Korea; 3 Department of Chemical and Biomolecular Engineering, Yonsei University, Seoul, 03722 Republic of Korea; 4 Center for Structural Biology, Center for Cancer Research, National Cancer Institute, Frederick, MD 21702 USA; 5 Samsung Display Co. LTD., Yongin-si, Gyeonggi-do 17113 Republic of Korea; 6 Institute of Engineering Research, College of Engineering, Seoul National University, Seoul, 08826 Republic of Korea; 7 Advanced Institutes of Convergence Technology, Seoul National University, Seoul, Gyeonggi-do 16229 Republic of Korea

**Keywords:** Materials science, Nanoscience and technology

## Abstract

Determining the 3D atomic structures of multi-element nanoparticles in their native liquid environment is crucial to understanding their physicochemical properties. Graphene liquid cell (GLC) TEM offers a platform to directly investigate nanoparticles in their solution phase. Moreover, exploiting high-resolution TEM images of single rotating nanoparticles in GLCs, 3D atomic structures of nanoparticles are reconstructed by a method called “Brownian one-particle reconstruction”. We here introduce a 3D atomic structure determination method for multi-element nanoparticle systems. The method, which is based on low-pass filtration and initial 3D model generation customized for different types of multi-element systems, enables reconstruction of high-resolution 3D Coulomb density maps for ordered and disordered multi-element systems and classification of the heteroatom type. Using high-resolution image datasets obtained from TEM simulations of PbSe, CdSe, and FePt nanoparticles that are structurally relaxed with first-principles calculations in the graphene liquid cell, we show that the types and positions of the constituent atoms are precisely determined with root mean square displacement values less than 24 pm. Our study suggests that it is possible to investigate the 3D atomic structures of synthesized multi-element nanoparticles in liquid phase.

## Introduction

Atomic structure analysis of colloidal nanoparticles is required for understanding and utilizing their distinctive chemical properties^1–3^. X-ray diffraction is used for a wide range of materials to investigate the fine structural characteristics^4–6^. X-ray absorption spectroscopy is also a powerful technique to acquire surface-related atomic structural information^7^. X-ray diffraction and X-ray absorption spectroscopy provide only ensemble information and cannot be used to analyze individual nanoparticle structures. Electron tomography can directly resolve 3D atomic structure of an individual nanoparticle by reconstructing a 3D density from tens of tilt series of STEM images, by which atomic structures of various nanoparticles with alloying, multiple twins, and amorphous phases are revealed^8,9^. However, a vacuum condition that is typically required to acquire for transmission electron microscopy (TEM) can lead to structural deformation of the nanoparticles^10^, which hampers precise determination of structure–property relationships^8,9,11–14^. In this respect, development of method to resolve 3D atomic structure in native condition is in growing importance^11,12^.

Brownian one-particle 3D reconstruction is a promising method to directly resolve the 3D atomic structure of nanoparticles in their native liquid environment^13–15^, which has an advantage to analyze their native structures in liquid media or their structural change under various reaction condition^16–18^. For examples, observing growth trajectories of colloidal nanoparticles with time-resolved 3D atomic structures allows us to understand the shape evolution mechanism depending on the synthetic condition. In Brownian one-particle reconstruction, 3D atomic structures are determined by estimating the 3D orientations of high-resolution 2D TEM images with different unknown projection directions of a rotating nanoparticle. Recently, Brownian one-particle reconstruction successfully revealed the 3D atomic structures of individual Pt nanoparticles with 19 pm precision^14^ and enabled detailed surface structure analysis of ligand-protected nanoparticles^19^. This method can be readily applied to investigate structural details of various metallic nanoparticles composed of a single element. However, many nanoparticles of importance for catalysis and opto-electronic applications have complex crystal structures and compositions^20,21^. A technical improvement is therefore needed to expand the application of Brownian one-particle reconstruction to reveal atomic structures of multi-element nanoparticles. For example, in disordered metallic alloy nanoparticles, surface atomic arrangement^22,23^ and strain induced by heteroatoms^24,25^ affect their catalytic activity and stability. Introduction of heteroatoms can induce different phases and domains within the nanoparticle, which tunes the catalytic properties of metallic alloy nanoparticles^26–28^. In quantum dot nanoparticles, the distortion of bond lengths and bond angles, the distribution of dopants, and the atomic arrangement at interfaces cause modification of their electronic structures^29,30^. Moreover, surface defects of quantum dots are strongly related to charge carrier trapping^30–33^. Atomic structure analysis through Brownian one-particle reconstruction has the potential to reveal the distribution of heteroatoms, how they induce atomic strain, how they distort the atomic bonds, and allow precise localization of defects in nanoparticles in the solution phase. However, one critical challenge is how to classify heteroatoms in the Coulomb density maps of multi-element nanoparticles obtained from Brownian one-particle reconstruction.

In this study, we introduce a 3D reconstruction strategy for multi-element nanoparticles. A workflow for obtaining 3D atomic structures for disordered and ordered multi-element nanoparticle systems is proposed, including image processing, initial 3D model preparation, low-pass filtration, and atom type classification. The 3D reconstruction process is simulated by reconstructing various multi-element nanoparticles from simulated 2D TEM images. The 3D reconstructions of geometrically optimized fcc FePt alloy, rocksalt PbSe, zinc blende CdSe, and wurtzite CdSe nanoparticles by density functional theory (DFT) calculations result in successful assignments of both atomic positions and types, showing that multi-element nanoparticles can be reconstructed, regardless of their phase, domain distribution, compositional randomness, or atomic number difference between the constituent atoms. Our method has the potential to expand the applicability of Brownian one-particle 3D reconstruction to multi-element nanoparticle systems.

### Strategies for multi-element nanoparticle 3D reconstruction

**Figure 1:**
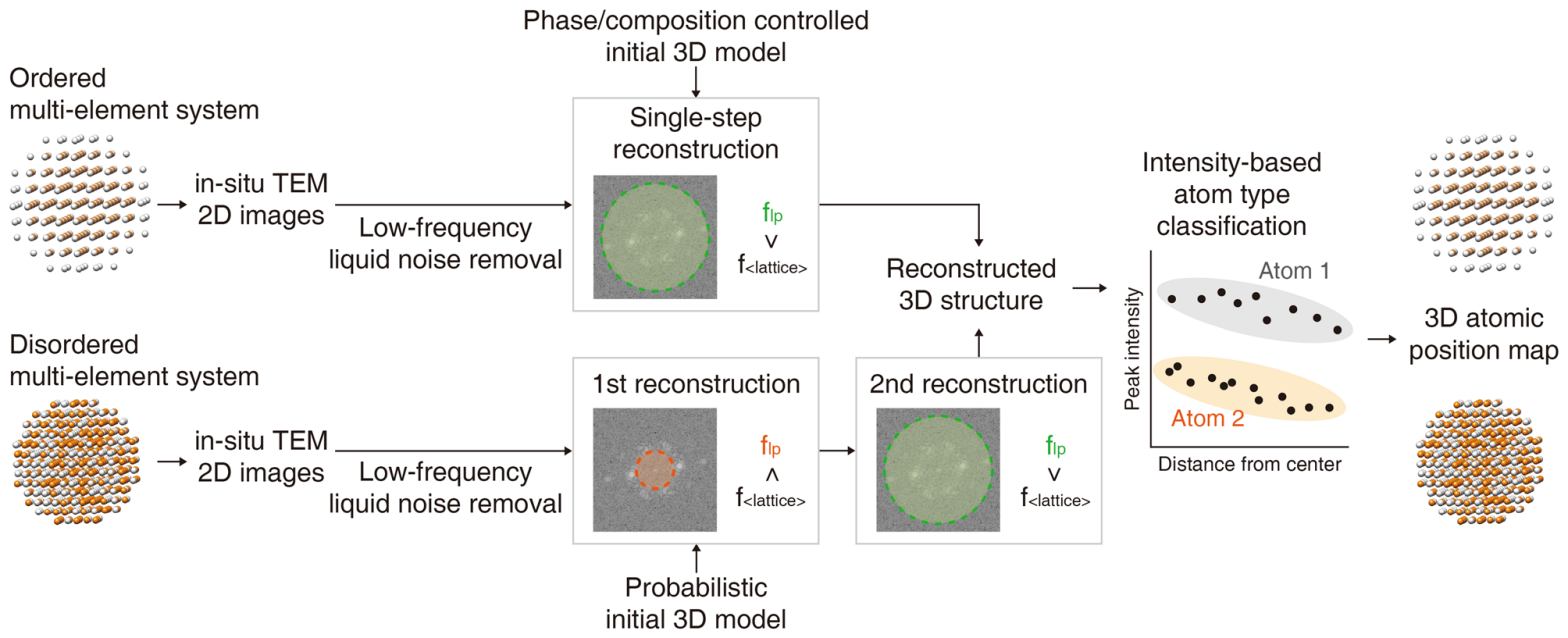
Overview of 3D reconstruction of multi-element nanoparticles. Ordered and disordered multi-element nanoparticles follow different pathways to obtain the atomic-resolution 3D structure. Low-frequency liquid noise is removed from the *in-situ* 2D TEM images first. Ordered multi-element systems are reconstructed through single-step reconstruction. The initial 3D model for ordered multi-element systems is phase and composition-controlled and the low-pass filter cutoff frequency includes the majority of the power spectrum peaks. Disordered multi-element systems are reconstructed through two-step reconstruction. The initial 3D model for disordered multi-element systems is obtained through probabilistic ab initio 3D reconstruction, with a low-pass limit cut-off frequency that vary depending on the stage of reconstruction (1st step: excluding lattice, 2nd step: including lattice). After the reconstruction, atom types are classified through analyzing the density variations in the reconstructed 3D density.

Reconstruction of the 3D atomic structures of multi-element nanoparticles requires strategies tailored to their structural characteristics. Ordered multi-element nanoparticles, such as wurtzite CdSe and rocksalt PbSe, have ordered lattice structures with periodic arrangements of the constituent atoms. Disordered multi-element nanoparticles also have a periodic lattice, but different types of atoms randomly occupy sites in the lattice. Examples of disordered multi-element nanoparticles include alloy FePt, preserving fcc-like crystal structure. The procedure for 3D reconstruction of ordered and disordered multi-element nanoparticles is summarized in Fig. 1.

As an initial step, low-frequency components originating from the liquid background are removed by averaging consecutive frames of *in-situ* TEM data, defined as moving averaging hereafter. Nanoparticles in consecutive frames of *in-situ* TEM data usually have very similar projection direction, thus moving averaging reduces the low-frequency liquid noise and enhances the signal to noise ratio (SNR). 3D reconstruction is initiated with an initial 3D model, and it guides finding the first relative 3D orientations of the 2D images that are subsequently refined. Different types of initial 3D models are used for ordered and disordered multi-element systems. In the ordered systems, an initial 3D model which has the same crystal structure and compositional arrangement as the targeting material is used. In the case of disordered systems, a probabilistic initial 3D model is generated by using PRIME^34^. Next, 3D reconstruction of the two different systems is conducted by different low-pass filtering strategies to control the frequency range used for matching reprojections of the 3D density with the 2D views. The low-pass filtering strategy for reconstructing the ordered systems is referred to as single-step reconstruction, since a single cut-off frequency of the low-pass filter is applied to include the majority of peaks in reciprocal space. Two-step reconstruction is suggested for 3D reconstruction of disordered multi-element systems. In the first step of two-step reconstruction, the cut-off frequency of the low-pass filter is set below the frequency of the peaks in reciprocal space, excluding lattice information. The second step utilizes lattice information by setting the cut-off frequency of the low-pass filter to include the peaks. Successful reconstruction of high-resolution 3D Coulomb density maps allows the 3D atomic positions to be assigned by identifying local maxima in the 3D density map. Plotting the intensity of the assigned atomic positions against the distance from the center of mass of the nanoparticle displays groups of points with different intensity, allowing classification of the different types of atoms.

**Figure 2:**
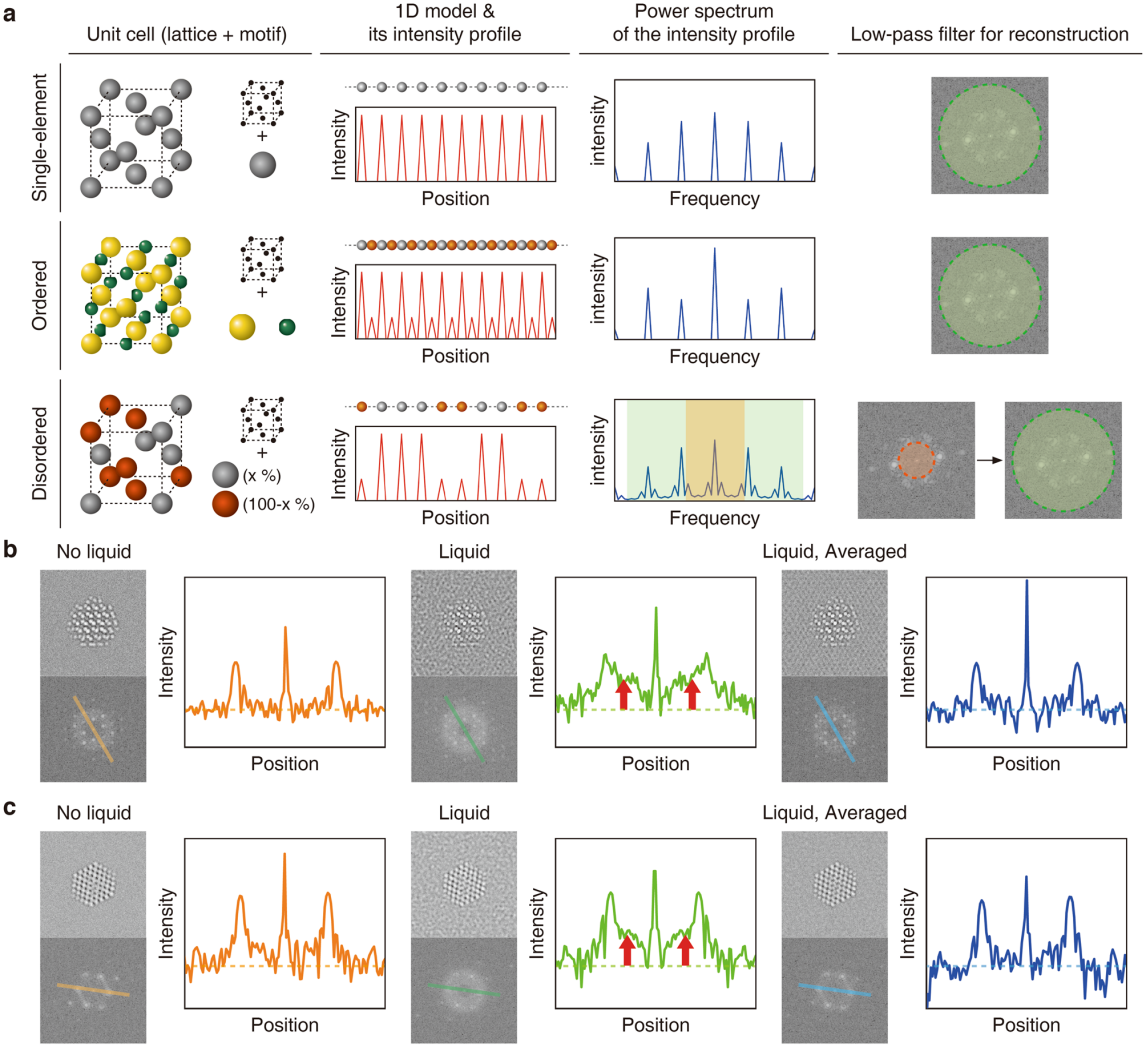
(**a**) Unit cell structures, 1D models and their intensity profiles, power spectrum of 1D modeled intensity profiles, and different low-pass limitation strategies for single-element, ordered multi-element, and disordered multi-element systems. (**b**–**c**) Simulated TEM images of multi-element nanoparticles, their 2D power spectra, and corresponding line profiles for nanoparticles without liquid (left, orange), with liquid (medium, green), and with liquid and averaging (right, blue). The line profiles are extracted from marked lines on the 2D power spectrum images. (**b**) ordered multi-element nanoparticles (zinc blende CdSe), (**c**): disordered multi-element nanoparticles (disordered fcc FePt).

3D reconstruction of multi-element nanoparticles is nontrivial, because the introduction of heteroatoms in the host crystal structure complicates the signal in reciprocal space. To understand the effect of the introduction of heteroatoms in the power spectrum of single-element, ordered multi-element, and disordered multi-element nanoparticles, we simulated those systems using 1D models constructed with the same period. From the 1D model systems, the intensity profile and power spectrum were obtained (Fig. 2a). As seen in the 1D intensity profiles and their power spectra, multi-element systems have additional information due to the distribution of heteroatoms, which is lacking in single-element systems.

The intensity profile of the 1D model for single-element systems shows a repeating array of peaks with identical intensity (Fig. 2a, top) and a power spectrum with a peak at frequency zero as well as additional peaks separated according to the reciprocal lattice parameter. The intensity profile of the 1D model for ordered multi-element systems, with a motif consisting of two elements, shows a repeating pattern of two peaks with different intensity (Fig. 2a, middle). The peak positions are the same as for the single-element systems, because the 1D models used in these two cases have the same periodicity. The power spectral density distributions of the two systems are expected to be different because the introduction of heteroatoms modifies the Fourier structure factors^35^. The 1D model for disordered multi-element systems has elements of two atomic species randomly distributed with uniform interatomic distance. It results in a richer intensity profile and power spectrum (Fig. 2a, bottom). We verified that introduction of heteroatoms modifies the low-frequency signal in 2D by comparing the TEM images of Pt and FePt nanoparticles obtained by experiment and simulation (Figs. S1 and S2), suggesting that careful experimentation with the low-pass frequency limit used in the initial stages of 3D reconstruction is likely to be as important as the production of an appropriate initial 3D model.

The initial 3D model is important for robust convergence of the 3D reconstruction process for multi- and single-element nanoparticles (Fig. 1). We argue that different initial 3D model approaches should be used for ordered and disordered multi-element systems. An initial 3D model embedding lattice information, which has the same phase and composition as the target material system, is suitable for 3D reconstruction of ordered multi-element systems^36^. For disordered multi-element systems, the situation is similar to that of 3D reconstruction of proteins by cryo-EM and single-particle analysis, where *ab-initio* initial 3D models are generated using a probabilistic approach^34^.

TEM images obtained from a GLC include significant background from the encapsulated liquid pocket, which needs to be reduced for successful 3D reconstruction. We compared nanoparticles contained in the GLC with those in vacuum through TEM simulation to study the effect of the low-frequency signal introduced by the GLC (Fig. 2b,c). The GLC creates a halo-ring effect in the low-frequency region, as shown in the power spectrum images of simulated TEM images and their corresponding line profiles (Fig. 2b,c). The intensity distribution of power spectrum peaks (ordered multi-element systems) or more complex signal distribution patterns (disordered multi-element systems) can be obscured by the low-frequency “noise” from the GLC. 3D structures reconstructed from simulated TEM images with GLC-induced low-frequency noise confirm that GLC-induced noise can interrupt high-resolution reconstruction (Figs. S3 and S4).

Averaging a series of TEM images that represents a nanoparticle, maintaining constant projection direction, in fluctuating liquid environment can be an efficient strategy to reduce GLC background noise. To validate this strategy, ten simulated TEM images of a nanoparticle with a fixed orientation were generated with ten different liquid coordinates and averaged into one image. Averaging diminishes GLC-induced low-frequency noise, as confirmed by the reduced intensity of the halo ring effect in the power spectrum of the averaged image (Fig. 2b,c). Moving averaging of *in-situ* TEM images is likely an effective way to remove undesirable GLC signals, considering that nanoparticles can maintain the same projection direction in a few consecutive *in-situ* TEM images. Moving averaging tends to increase the intensity of both low-frequency noise and nanoparticle signal (Fig. S5). The degree of signal enhancement is much greater for the nanoparticle signal compared to the low-frequency noise, making the undesired GLC signals suppressed (Fig. S5). In the experimental application, it is required to optimize moving averaging depending on the nature of the rotating nanoparticle in the liquid cell, which is affected by the type and thickness of the liquid, surface interactions, and the signal-to-noise ratio. It is not desired to average image frames including different orientations of the particle. Beam-induced anisotropic motion is also commonly used for *in-situ* TEM images and weighted time window averaging in conjunction with anisotropic motion correction will further assist in the successful 3D reconstruction of multi-element nanoparticles from experimental data^36,37^.

### Test results

**Figure 3:**
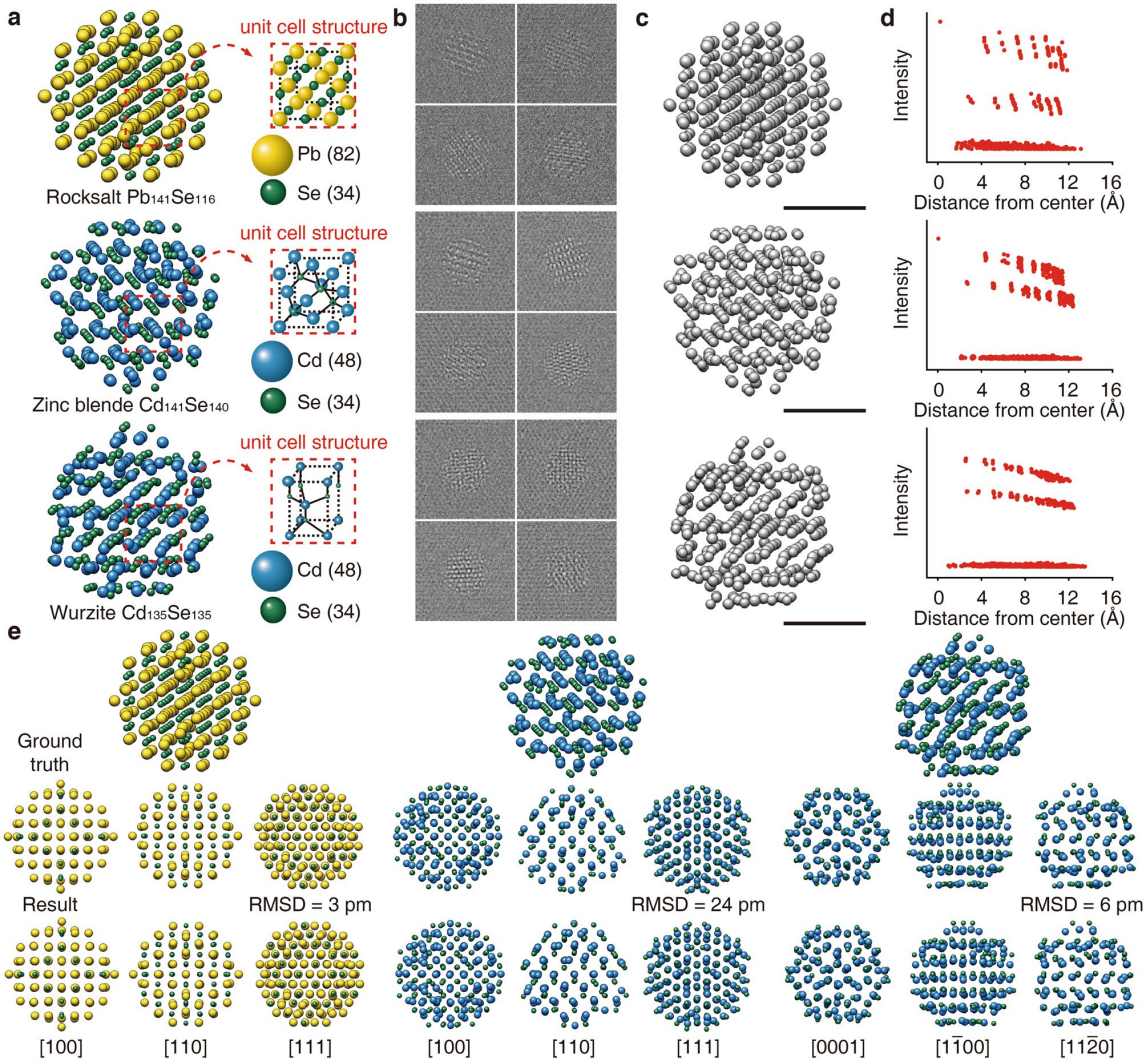
3D reconstruction process for ordered multi-element nanoparticles. (top row: rocksalt PbSe, middle row: zinc blende CdSe, bottom row: wurtzite CdSe) (**a**) Ground truth atomic structures. Yellow, green, and blue spheres correspond to Pb, Se, and Cd atoms, respectively. Ground truth structures are geometrically optimized by DFT calculations. (**b**) Representative simulated TEM images. (**c**) 3D Coulomb density maps obtained by single-step reconstruction. (**d**) Plots of distance from center of mass as a function of local maximal intensity. (**e**) Atomic structures obtained from 3D Coulomb density maps with atom type classification. The reconstructed atomic structures are compared with the ground truth structures along different zone axes. Root mean square displacement (RMSD) between input and reconstructed structures are depicted. Scale bars, 1 nm.

PbSe (rocksalt) and CdSe (wurtzite and zinc blende) nanoparticles with 2.5 nm in diameter were used to demonstrate 3D reconstruction of ordered multi-element systems. Three types of nanoparticles were geometrically optimized by DFT calculations to account for realistic nanoparticle structures that include slight structural deviations from the bulk counterparts (Fig. 3a). We generated 50,000 TEM images by multislice simulation^38^ (5000 projection directions × 10 different liquid coordinates/projection direction) and averaged 10 images with the same projection directions to reduce liquid noise effects, providing 5000 images for 3D reconstruction (Fig. 3b).

The ground truth structures of the nanoparticles were rotated in a 50 × 50 × 100 Å supercell, resulting in 5000 coordinates with different projection directions. To simulate GLC environments, coordinates for single-layer graphene were added to the top and bottom of the supercell. A few hundred sets of coordinates for toluene molecules were generated by molecular dynamics simulation of a supercell with an equivalent size containing toluene molecules. Ten sets of coordinates were randomly selected for each projection direction (details of the molecular dynamics simulation are in "Methods" section). As-prepared toluene solvent molecules were placed with the nanoparticle between the graphene layers. From the 50,000 generated coordinates of the nanoparticle in GLC, 50,000 TEM images were produced by multislice TEM simulation (see Methods). Each set of 10 images with the same projection directions of the nanoparticle was averaged into one image. Gaussian noise was added to the images to account for instrumental noise sources in TEM imaging. The noise-added images have lower SNR than the experimentally acquired TEM images (see Table S1). The resulting 5000 averaged images were used as input for 3D reconstruction.

An initial 3D model with the same crystal structure and compositional arrangement as the target material was used for 3D reconstruction. A low-pass filter cutoff threshold of (1 Å)^−1^ was used, including all noticeable power spectrum peaks (Table S2). The SINGLE suite was used for 3D reconstruction (details of parameters are in Methods)^36^. The obtained 3D Coulomb density maps from the single-step reconstruction are shown in Fig. 3c. Please note that the single-step method can be also used for reconstructing single-component nanoparticles such as Pt nanoparticles, as shown in the 3D coulomb density map obtained from the single-step reconstruction of experimental GLC data of a Pt nanoparticle adopted form the previous publication (Fig. S6)^14^. Intensity distributions of local maxima show that three groups, one for Pb(Cd) atom (top group), another for Se atom (middle group), and the other for noise (bottom group), are distinguished, as shown in Fig. 3d. The number of points for each group is the same as that of the corresponding element in the input structure without missing a single atom. Comparing the reconstructed atomic structures with the input atomic structures along various directions shows that not only the number of atoms but their positions are accurately reconstructed (Fig. 3e). Root mean square displacement (RMSD) values between the input structures and the reconstructed structures are less than 24 pm, indicating precise reconstruction of atomic positions. Slices through the 3D atomic maps confirm successful 3D reconstruction (Fig. S7). Additionally, we evaluated the 3D reconstruction results by projection-reprojection comparison and coverage of assigned projection directions (see Fig. S8). The reprojected images from the 3D Coulomb density maps agree well with the original projection images. The distribution of the assigned projection direction shows a broad coverage in 3D space, as expected. We also confirmed that the single-step method is more effective for the ordered multi-element systems compared to the two-step method (Fig. S9). For successful reconstruction of ordered multi-element nanoparticles using the single-step method, optimization of the low-pass filter cutoff frequency is necessary. Using a more aggressive low-pass boundary reduces the computational cost, but the quality of 3D reconstruction decreases, or the reconstruction process may fail (Fig. S10). A 3 nm-sized PbSe nanoparticle was also successfully reconstructed under the same conditions as used for the 2.5 nm-sized nanoparticles (Fig. S11), indicating that the suggested 3D reconstruction method can be extended to larger nanoparticles.

**Figure 4:**
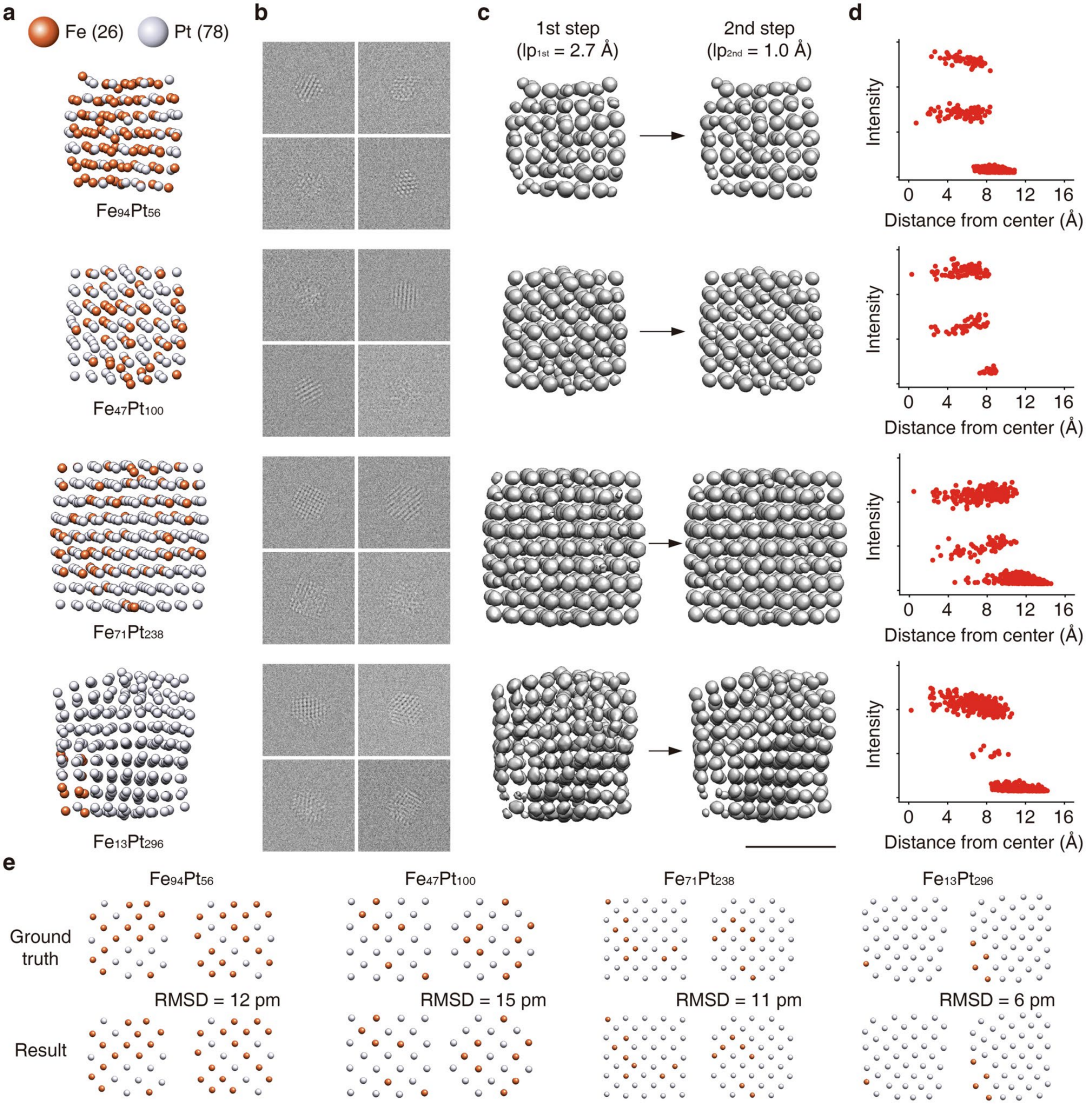
3D reconstruction process for disordered multi-element nanoparticles. (**a**) Input atomic structures. Grey and orange spheres correspond to Pt and Fe atoms, respectively. Ground truth structures are geometrically optimized by DFT calculations. (**b**) Representative simulated TEM images. (**c**) 3D Coulomb density maps obtained by two-step reconstruction. (**d**) Plots of distance from center of mass as a function of local maximal intensity. (**e**) Representative slices of 3D atomic maps of ground truth structures and reconstructed structures. Root mean square displacement (RMSD) between input and reconstructed structures are depicted. Scale bar, 1 nm.

Four DFT-relaxed random fcc FePt nanoparticles with different size, composition and structural disorder were used to demonstrate 3D reconstruction of disordered multi-element systems (Fig. 4a). We generated TEM images of FePt nanoparticles in the same way as in the ordered nanoparticle cases, mentioned above (Fig. 4b). 3D structures of four FePt nanoparticles were reconstructed by two-step reconstruction. Cutoff low-pass thresholds of (2.7 Å)^−1^ and (1.0 Å)^−1^ were consecutively used for the first and second steps (Fig. 4c). The threshold of (2.7 Å)^−1^ is lower than the lowest frequency among the reciprocal peaks for fcc Pt (d_Pt(111)_ = 2.296 Å) in the first step. In the second step, a threshold of (1.0 Å)^−1^ was applied to include the majority of peaks. The obtained 3D Coulomb density maps from the two-step reconstruction are shown in Fig. 4c. Intensity distributions of local maxima show that the points are distinctly divided into 3 groups, which are Pt (top), Fe (middle), and noise (bottom) groups, as shown in Fig. 4d. Both type and position of the constituent atoms are precisely reconstructed (Fig. 4d,e, and Fig. S12). The reconstructed atomic structures are in good agreement with the input structures, with RMSD values less than 15 pm. Although the low-pass limit employed in the first step completely excludes the reciprocal peaks, the 3D Coulomb density maps from the first step were similar to the input structures, indicating that there is enough low-frequency signal to guide projection direction assignment (Fig. 4c). To further validate the 3D reconstruction results, we confirmed that the reprojected images from the 3D volume maps matched the original projection images. Assigned projection directions had wide coverages in 3D space (Fig. S13). Notably, the single-step reconstruction fails to reconstruct the 3D structure of disordered FePt nanoparticles (Fig. S14). Successful reconstruction of disordered multi-element nanoparticles requires careful optimization of the low-pass cutoff frequency in the first step. The low-pass limit for the first step should include as much information as possible while excluding strong reciprocal peaks. Either a low-pass filter including strong peaks ((2 Å)^−1^) or a low-pass filter removing too much information ((4 Å)^−1^) leads to poor reconstruction results (Fig. S15). By using two-step reconstruction with careful optimization of the low-pass limit, the 3D atomic structures of disordered multi-element nanoparticles can be reconstructed. We also confirmed that two-step reconstruction can be extended to larger nanoparticles by reconstructing 3 nm-sized FePt nanoparticles (Fig. S16). The suggested 3D reconstruction strategy is robust with different solvent types. For example, toluene and water, when used as a solvent, show differences in the position, thickness, and intensity of halo-ring (Fig. S17). However, with an optimized moving averaging, it was demonstrated that PbSe and FePt are successfully reconstructed both in toluene and water (Figs. 3, 4, and S17).

## Discussion

In this report, we present a strategy to reconstruct the 3D atomic structures of multi-element nanoparticles in liquid through Brownian one-particle reconstruction. Different reconstruction strategies are proposed for ordered and disordered multi-element nanoparticles, which is supplemented by highly reliable database using first-principles calculations. Single-step reconstruction is effective for 3D reconstruction of ordered multi-element nanoparticles, as demonstrated with rocksalt PbSe, zinc blende CdSe, and wurtzite CdSe. 3D reconstruction of disordered multi-element nanoparticles can be conducted by two-step reconstruction, as seen in the 3D reconstruction of four disordered fcc FePt nanoparticles. There is no doubt that the 3D reconstruction of synthesized multi-element nanoparticles, using images obtained from experimental liquid-phase TEM, will be more challenging than described here and may need further improvement in the 3D reconstruction methodology. Notably, our simulations do not account for the isotropic and anisotropic atomic displacements that are present in experimental data, which cause a strong radial dependency of the atom intensity distribution in experimentally obtained 3D reconstructions. In real-world 3D reconstructions, the atom intensity is strong in the ordered core and decreases as we move closer to the surface, where the coordination is lower, solvent effects are prominent, surface adsorbed species exist, and atom mobility is high. It is possible that radial normalization would be needed to correctly identify the right atomic species, or better yet, the atomic displacement parameters could be derived from the experimental density maps and a probabilistic framework that models the atomic displacements and determines a probability rather than a deterministic assignment of the atomic species could be built. This is the subject of our future research, and we foresee that there will be an ordered core of atoms for which the species of the elements can be readily determined, but when atomic displacements dominate the intensity profile close the surface, it will be very difficult to determine the atomic species, especially if the atomic number difference between the different elements is small. Nonetheless, the considerations and procedures introduced in this report can be a practical guidance toward structural investigation of individual multi-element nanoparticles in 3D space.

## Methods

### DFT calculation for alloy NPs

Density functional theory (DFT) calculations were performed by Vienna ab-initio simulation package (VASP)^39^ with the projector-augmented wave (PAW)^40^ pseudopotentials. The exchange–correlation functional of Perdew-Burke Ernzerhof (PBE)^40^ generalized gradient approximation (GGA)^41^ was adopted. To obtain the structure change of multi-component trajectories, we optimized several binary nanoparticles (~ 2.5 nm). Spin polarization was considered, and the basis plane waves were expanded with cutoff energy of 400 eV. Only Γ-point scheme was used for the nanoparticle calculations, and the vacuum space was imposed to secure 10 Å within the periodic images for each system. Structures were relaxed until the energy and force convergences of 10^–5^ eV and 0.05 eV⋅Å^−1^, respectively.

### Details of TEM image simulation

Simulated 50,000 TEM images for DFT-relaxed rocksalt PbSe, zinc blende CdSe, wurtzite Cdse, and disordered fcc FePt nanoparticles (with sizes of 2.5 nm for ordered systems and 1.5–2.0 nm for disordered systems) were obtained by randomly rotating the given DFT-relaxed coordinate for each nanoparticle 5000 times with 10 different solution coordinates for each rotation within a 50.00 Å (200 pixels × 0.25 Å/pixel) super cell (as mentioned in the body). The super cells were split into multiple 1.0-Å-thick slices along the z-axis, with 200 × 200 pixels sampling in the x and y axis. The following input parameters were used for the simulations: acceleration voltage of 200 kV, mean, standard deviation and sampling size of defocus distribution of 60, 40 and 10 Å, C_3_ aberration of − 0.01 mm, C_5_ aberration of 3 mm, objective aperture of 30 mrad, minimum and maximum illumination angle of 0 and 1 mrad, thermal temperature of 273 K thermal, and a number or thermal vibration configurations of 5.

### MD simulation for solvent coordinates

Toluene molecules and graphene sheets were introduced to reproduce a more realistic liquid condition. In supercell, coordinates for single-layer graphene coordinates are located on the top and bottom of water layer with a thickness of 10 nm. The atomic coordinates of toluene were obtained by a classical molecular dynamics simulation using the Large-scale Atomic/Molecular Massively Parallel Simulator (LAMMPS) package. A CGenFF force field was used for the simulation. 6000 toluene molecules were randomly generated in the 50.00 Å × 50.00 Å × 100.0 Å supercell. The simulation was conducted with the energy minimization of the system as an initial condition. NVT ensemble, including 1 fs time step and Nosé–Hoover thermostat, was performed for 100 ps and toluene trajectories were recorded every 100 fs. TEM images of the system including nanoparticle, toluene, and graphene were obtained through multi-slice simulation. Simulated TEM image set reflects random rotation of nanoparticles and fluctuations of toluene molecules in the GLC. Atomic displacements within the nanoparticles themselves were not modeled. TEM images, adding Gaussian noise with a signal-to-noise ratio of 0.5, were used for 3D reconstruction.

### Detailed parameters and procedures of 3D reconstructions

The 5000 averaged TEM images for each nanoparticle were statistically normalized to have an average of 0 and unit pixel variance. The image contrast was inverted, which is necessary for the reconstruction method, which assumes white atom contrast. Gaussian noise was added to emulate TEM instrumentation noise, including shot noise due to the quantum nature of electron scattering and detector noise. These pre-processed stacks of images were used as input for reconstruction using the SINGLE collection of algorithms^36^. The following input parameters were used in SINGLE. 3D structures of one PbSe nanoparticle and two CdSe nanoparticles were reconstructed using the single-step approach. In the single-step method, spherically truncated initial 3D models with the same lattice structure as the corresponding nanoparticles were used. In the first step of the single-step approach, the low-pass limit was set to (1.0 Å)^−1^, and the number of projection directions (parameter *nspace*) was 5000. In the following refinement step, the values were set to (1.0 Å)^−1^ and 10,000, respectively. 3D structures of four FePt nanoparticles were reconstructed using the two-step approach. The PRIME algorithm^34^ was used in conjunction with a 2.7 Angstrom low-pass limit and 3000 projection direction candidates to generate an initial 3D model. In the first step of the two-step approach (PRIME), the low-pass limit was set to (2.7 Å)^−1^, and the number of projection directions (parameter *nspace*) was 3000. In the following refinement step, the values were set to (1.0 Å)^−1^ and 10,000, respectively.

## Supplementary Information

Supplementary Information.

## Data Availability

The datasets used and/or analysed during the current study available from the corresponding author on reasonable request.

## References

[1] Zhang J (2021). Atomic scale tracking of single layer oxide formation: Self-peeling and phase transition in solution. Small Methods.

[2] Xiao L (2021). Engineering of amorphous PtOx interface on Pt/WO3 nanosheets for ethanol oxidation electrocatalysis. Adv. Funct. Mater..

[3] An H (2022). Mechanism and performance relevance of nanomorphogenesis in polyamide films revealed by quantitative 3D imaging and machine learning. Sci. Adv..

[4] Castillo-Blas C, Moreno JM, Romero-Muñiz I, Platero-Prats AE (2020). Applications of pair distribution function analyses to the emerging field of non-ideal metal–organic framework materials. Nanoscale.

[5] Yan N (2018). Unraveling the long-pursued Au144 structure by X-ray crystallography. Sci. Adv..

[6] Nath D, Singh F, Das R (2020). X-ray diffraction analysis by Williamson–Hall, Halder–Wagner and size-strain plot methods of CdSe nanoparticles- a comparative study. Mater. Chem. Phys..

[7] Song S (2018). Operando X-ray spectroscopic tracking of self-reconstruction for anchored nanoparticles as high-performance electrocatalysts towards oxygen evolution. Energy Environ. Sci..

[8] Chen CC (2013). Three-dimensional imaging of dislocations in a nanoparticle at atomic resolution. Nature.

[9] Yang Y (2017). Deciphering chemical order/disorder and material properties at the single-atom level. Nature.

[10] Albrecht W (2019). Thermal stability of gold/palladium octopods studied in situ in 3D: Understanding design rules for thermally stable metal nanoparticles. ACS Nano.

[11] Xia W (2018). Bimetallic nanoparticle oxidation in three dimensions by chemically sensitive electron tomography and in situ transmission electron microscopy. ACS Nano.

[12] Koneti S (2019). Fast electron tomography: Applications to beam sensitive samples and in situ TEM or operando environmental TEM studies. Mater Charact..

[13] Kim BH, Heo J, Park J (2021). Determination of the 3D atomic structures of nanoparticles. Small Sci..

[14] Kim BH (2020). Critical differences in 3D atomic structure of individual ligand-protected nanocrystals in solution. Science.

[15] Park J (2015). 3D structure of individual nanocrystals in solution by electron microscopy. Science.

[16] Ye X (2016). Single-particle mapping of nonequilibrium nanocrystal transformations. Science.

[17] Ma X, Lin F, Chen X, Jin C (2020). Unveiling growth pathways of multiply twinned gold nanoparticles by in situ liquid cell transmission electron microscopy. ACS Nano.

[18] Liao HG (2014). Facet development during platinum nanocube growth. Science.

[19] Kim S (2021). Correlating 3D surface atomic structure and catalytic activities of Pt nanocrystals. Nano Lett..

[20] Casar JR, McLellan CA, Siefe C, Dionne JA (2021). Lanthanide-based nanosensors: Refining nanoparticle responsiveness for single particle imaging of stimuli. ACS Photon..

[21] Lay A (2017). Upconverting nanoparticles as optical sensors of nano- to micro-newton forces. Nano Lett..

[22] Chung YH (2014). Effect of surface composition of Pt–Fe nanoparticles for oxygen reduction reactions. Int. J. Hydrog. Energy.

[23] Chen W, Kim J, Sun S, Chen S (2008). Electrocatalytic reduction of oxygen by FePt alloy nanoparticles. J. Phys. Chem. C.

[24] Escudero-Escribano M (2016). Tuning the activity of Pt alloy electrocatalysts by means of the lanthanide contraction. Science.

[25] Strasser P (2010). Lattice-strain control of the activity in dealloyed core–shell fuel cell catalysts. Nat. Chem..

[26] Jung WS, Popov BN (2017). Effect of pretreatment on durability of fct-structured Pt-based alloy catalyst for the oxygen reduction reaction under operating conditions in polymer electrolyte membrane fuel cells. ACS Sustain. Chem. Eng..

[27] Kong F (2020). Active and stable Pt-Ni Alloy octahedra catalyst for oxygen reduction via near-surface atomical engineering. ACS Catal..

[28] Zheng L (2020). A unique pathway of PtNi nanoparticle formation observed with liquid cell transmission electron microscopy. Nanoscale.

[29] Anderson NC, Hendricks MP, Choi JJ, Owen JS (2013). Ligand exchange and the stoichiometry of metal chalcogenide nanocrystals: Spectroscopic observation of facile metal-carboxylate displacement and binding. J. Am. Chem. Soc..

[30] Kirkwood N (2018). Finding and fixing traps in II–VI and III–V colloidal quantum dots: The importance of Z-type ligand passivation. J. Am. Chem. Soc..

[31] Eagle FW, Park N, Cash M, Cossairt BM (2021). Surface chemistry and quantum dot luminescence: Shell growth, atomistic modification, and beyond. ACS Energy Lett..

[32] Houtepen AJ, Hens Z, Owen JS, Infante I (2017). On the origin of surface traps in colloidal II–VI semiconductor nanocrystals. Chem. Mater..

[33] Giansante C, Infante I (2017). Surface traps in colloidal quantum dots: A combined experimental and theoretical perspective. J. Phys. Chem. Lett..

[34] Elmlund H, Elmlund D, Bengio S (2013). PRIME: Probabilistic initial 3D model generation for single-particle cryo-electron microscopy. Structure.

[35] Zou X, Hovmöller S, Oleynikov P (2011). Electron Crystallography: Electron Microscopy and Electron Diffraction.

[36] Reboul CF (2021). SINGLE: Atomic-resolution structure identification of nanocrystals by graphene liquid cell EM. Sci Adv.

[37] Zheng SQ (2017). MotionCor2: Anisotropic correction of beam-induced motion for improved cryo-electron microscopy. Nat. Methods.

[38] Kirkland EJ (2010). Advanced Computing in Electron Microscopy.

[39] Kresse G, Furthmüller J (1996). Efficiency of ab-initio total energy calculations for metals and semiconductors using a plane-wave basis set. Comput. Mater Sci..

[40] Blöchl PE (1994). Projector augmented-wave method. Phys. Rev. B.

[41] Perdew JP, Burke K, Ernzerhof M (1996). Generalized gradient approximation made simple. Phys. Rev. Lett..

