# Supplementary material for: Method for 3D atomic structure determination of multi-element nanoparticles with graphene liquid-cell TEM
Source: Sci Rep. 2023 Feb 1;13:1814. doi: 10.1038/s41598-023-28492-5 (PMC9892495; doi:10.1038/s41598-023-28492-5)
Supplement: Supplementary file 1 — Supplementary Information. [file 41598_2023_28492_MOESM1_ESM.pdf]

Supporting information

Method for 3D atomic structure determination of multi-element nanoparticles with graphene liquid-cell TEM

Junyoung Heo[#1,2], Dongjun Kim[#1], Hyesung Choi[#1], Sungin Kim[1,2], Hoje Chun[3], Cyril F. Reboul[4], Cong T. S. Van[4], Dominika Elmlund[4], Soonmi Choi[5], Kihyun Kim[5], Younggil Park[5], Hans Elmlund[4], Byungchan Han[3] & Jungwon Park[*1,2,6,7]

1 School of Chemical and Biological Engineering, and Institute of Chemical Processes
Seoul National University
Seoul 08826, Republic of Korea
2 Center for Nanoparticle Research
Institute for Basic Science (IBS)
Seoul 08826, Republic of Korea
3 Department of Chemical & Biomolecular Engineering
Yonsei University
Seoul 03722, Republic of Korea
4 Center for Structural Biology
Center for Cancer Research, National Cancer Institute
Frederick, MD 21702, USA
5 Samsung Display Co. LTD.
Gyeonggi-do 17113, Republic of Korea
6 Institute of Engineering Research, College of Engineering
Seoul National University
Seoul 08826, Republic of Korea
7 Advanced Institutes of Convergence Technology
Seoul National University
Gyeonggi-do 16229, Republic of Korea
* Corresponding Authors: hans.elmlund@nih.gov, bchan@yonsei.ac.kr, jungwonpark@snu.ac.kr
# Contributed equally

Particle	Method	System	Average SNR (and standard deviation)
1st particle in Figure 3	Simulation (multislice)	PbSe (rocksalt)	0.0302 (±0.0102)
2nd particle in Figure 3	Simulation (multislice)	CdSe (zinc blende)	0.0308 (±0.0092)
3rd particle in Figure 3	Simulation (multislice)	CdSe (wurtzite)	0.0319 (±0.0093)
1st particle in Figure 4	Simulation (multislice)	FePt (disordered fcc)	0.0287 (±0.0144)
2nd particle in Figure 4	Simulation (multislice)	FePt (disordered fcc)	0.0401 (±0.0157)
3rd particle in Figure 4	Simulation (multislice)	FePt (disordered fcc)	0.0272 (±0.0115)
4th particle in Figure 4	Simulation (multislice)	FePt (disordered fcc)	0.0317 (±0.0113)
Particle 1 in Ref. 15	Experiment (*in-situ* TEM)	Pt (fcc)	0.0539 (±0.0190)
Particle 4 in Ref. 15	Experiment (*in-situ* TEM)	Pt (fcc)	0.0444 (±0.0144)

Table S1. Signal-to-noise ratio (SNR) of simulated images used for demonstration and experimentally acquired images.

Rocksalt PbSe									
2theta (°)	24.78	28.69	41.021	48.517	50.824	59.407	65.364	67.284	74.728
d-space (Å)	3.593	3.112	2.2	1.876	1.796	1.556	1.428	1.392	1.27
hkl	111	200	220	311	222	400	331	420	422
Relative intensity in XRD	35.537	100	77.422	19.333	27.891	13.163	8.252	36.536	27.344
Wurtzite CdSe									
2theta (°)	23.377	24.834	26.518	34.367	41.085	44.777	47.805	48.603	49.569
d-space (Å)	3.805	3.585	3.361	2.609	2.197	2.024	1.903	1.873	1.839
hkl	$10\bar{1}0$	0002	$10\bar{1}1$	$10\bar{1}2$	$2\bar{1}\bar{1}0$	$10\bar{1}3$	$20\bar{2}0$	$2\bar{1}\bar{1}2$	20-21
Relative intensity in XRD	100	59.859	75.244	38.383	88.746	88.042	14.135	56.082	13.033
Zinc blende CdSe									
2theta (°)	24.822	28.739	41.093	48.604	50.915	59.516	65.486	67.411	74.874
d-space (Å)	3.587	3.106	2.197	1.873	1.793	1.553	1.425	1.389	1.268
hkl	111	200	220	311	222	400	331	420	422
Relative intensity in XRD	100	3.248	74.804	46.789	0.829	12.092	18.515	0.957	24.287

Table S2. D-space values of rocksalt PbSe, wurtzite CdSe, and zinc blende CdSe.

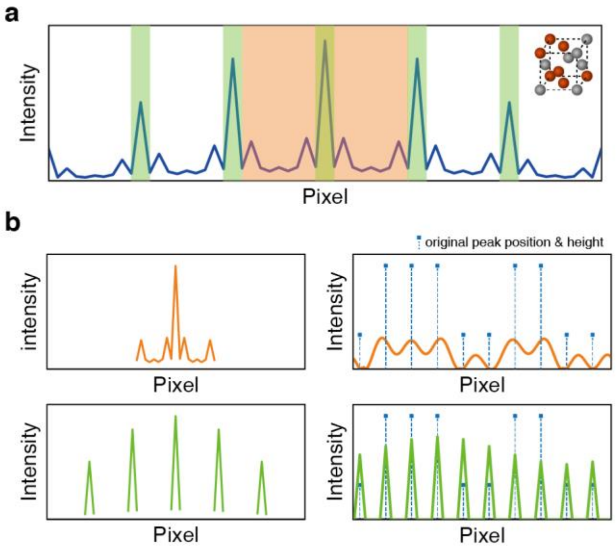

Figure S1. Inverse FFT of separated FFT of 1D modeled intensity profile of disordered multi-element systems. (a) FFT of 1D modeled intensity profile with color marks (orange: low-frequency background signals, green: major peaks from lattice structure). (b) Inverse FFTs of low-frequency background signals (top, orange) and major peaks corresponding lattice (bottom, green). Original peak positions and heights are displayed with cyan color.

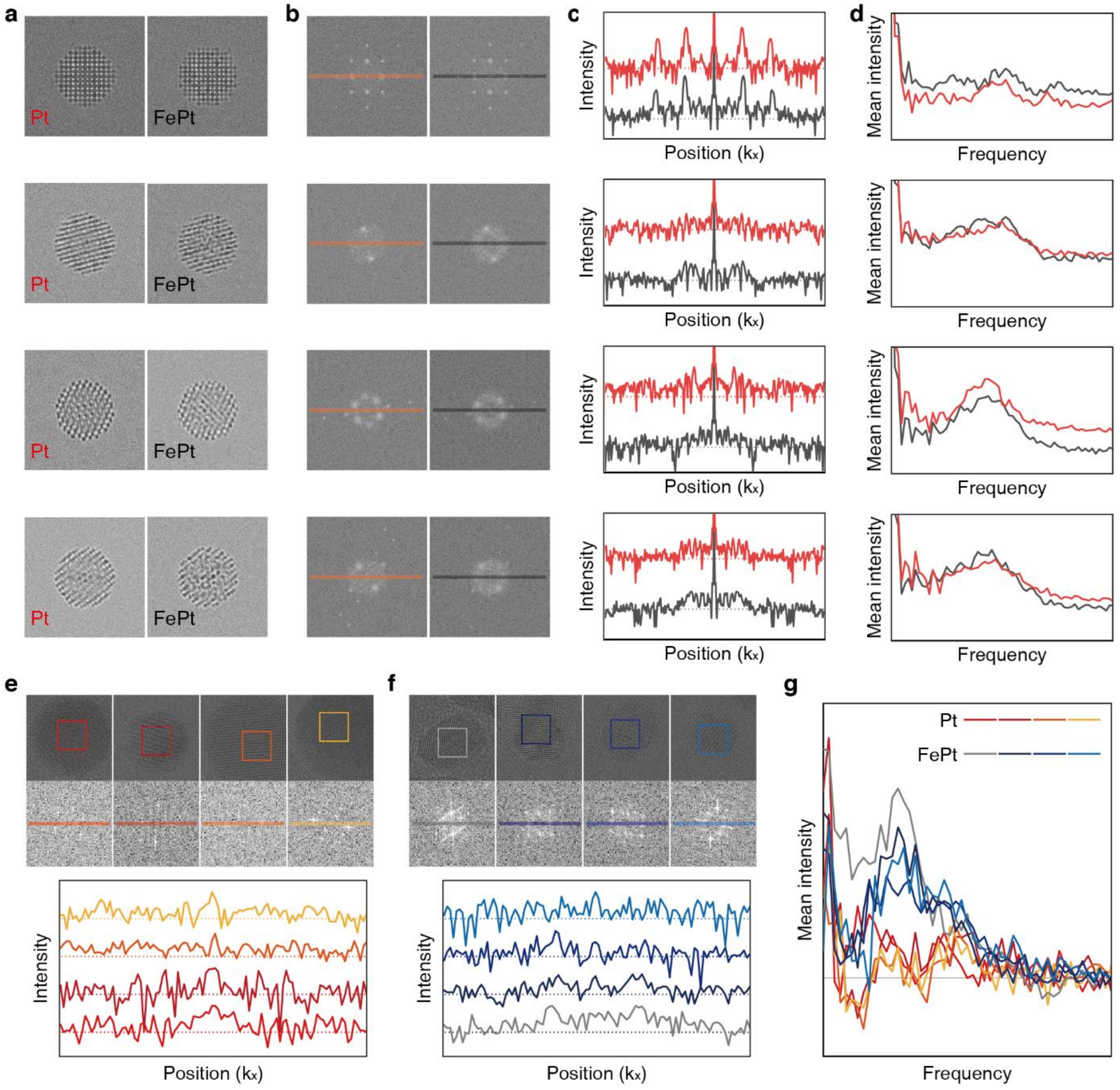

Figure S2. Comparison between TEM images of fcc Pt and disordered-fcc FePt. (a) Simulated TEM images of Pt and FePt nanoparticles with different projection directions. (b) 2D power spectrum images from the simulated TEM images. (c-d) Line profiles and Radial profiles of power spectrum images from Pt nanoparticle (red) and FePt nanoparticle (black). (e-f) Experimental TEM images of Pt and FePt nanoparticles, their power spectrum images from selected area, and corresponding line profiles. 6-10 nm-sized Pt and FePt nanoparticles are synthesized[45,46], followed by ligand removal using $NOBF_4$[47]. For synthesizing Pt nanoparticles, trace amount of $Fe(CO)_5$ is not added. Ligand-removed Pt and FePt nanoparticles placed on graphene-coated TEM grid[48]. TEM images are acquired by using JEM-ARM200F equipped with spherical-aberration corrector and K3 IS direct electron detector. (g) Radial profiles of power spectrum images from experimental TEM images.

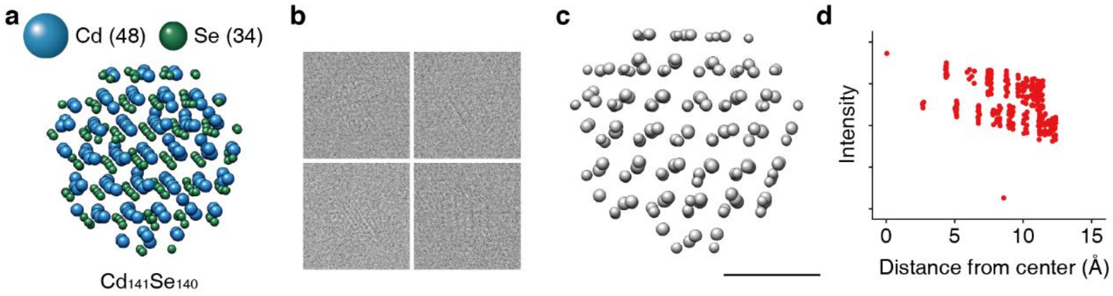

Figure S3. 3D reconstruction of ordered multi-element nanoparticle using simulated TEM images without liquid noise removal. (a) Ground truth atomic structure of geometrically optimized CdSe nanoparticle. (b) Representative simulated TEM images. (c) The resulting 3D Coulomb density map. (d) The plot of local maximal intensity as a function of distance from the center of mass. The types of atoms cannot be classified, even if the positions of the atoms are well assigned.

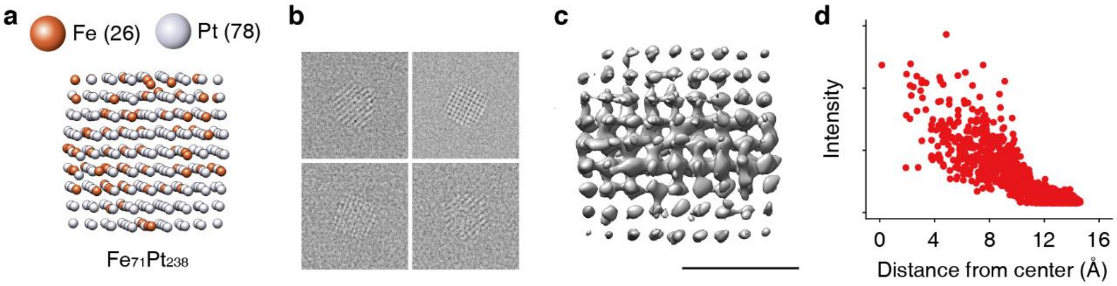

Figure S4. 3D reconstruction of disordered multi-element nanoparticle using simulated TEM images without liquid noise removal. (a) Input atomic structures of geometrically optimized FePt nanoparticle. (b) Representative simulated TEM images. (c) The resulting 3D Coulomb density map. (d) The distance from the center of mass plotted as a function of local maximal intensity. 3D reconstruction using simulated TEM images of disordered fcc FePt with GLC-induced noise results in a poor 3D Coulomb density map and atom types cannot be classified.

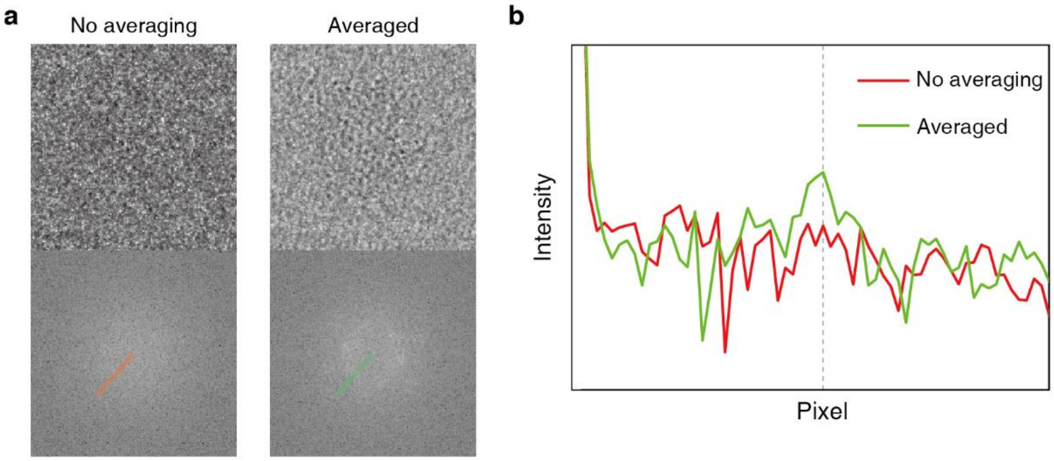

Figure S5. Effect of moving averaging to experimental LP-TEM data. TEM images of nanoparticles in GLC without averaging and with averaging are compared each other. (a) Representative TEM images of zinc blende CdSe nanoparticles in toluene solvent and their FFT pattern without averaging (right) and with averaging (left). (inset: TEM image) (b) Line profiles of FFT images. Dashed line indicates peak position which corresponds to CdSe (220) lattice structure.

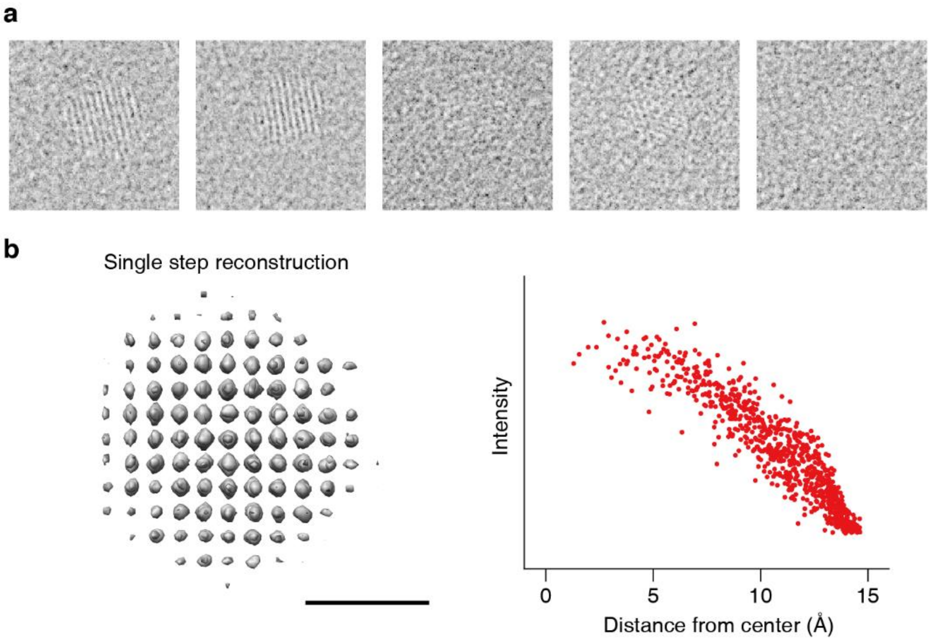

Figure S6. 3D reconstruction results using LP-TEM data of Pt nanoparticle. (a) Representative images of time-series LP-TEM data. (b) 3D reconstruction result by using single step reconstruction. Scale bar, 1 nm.

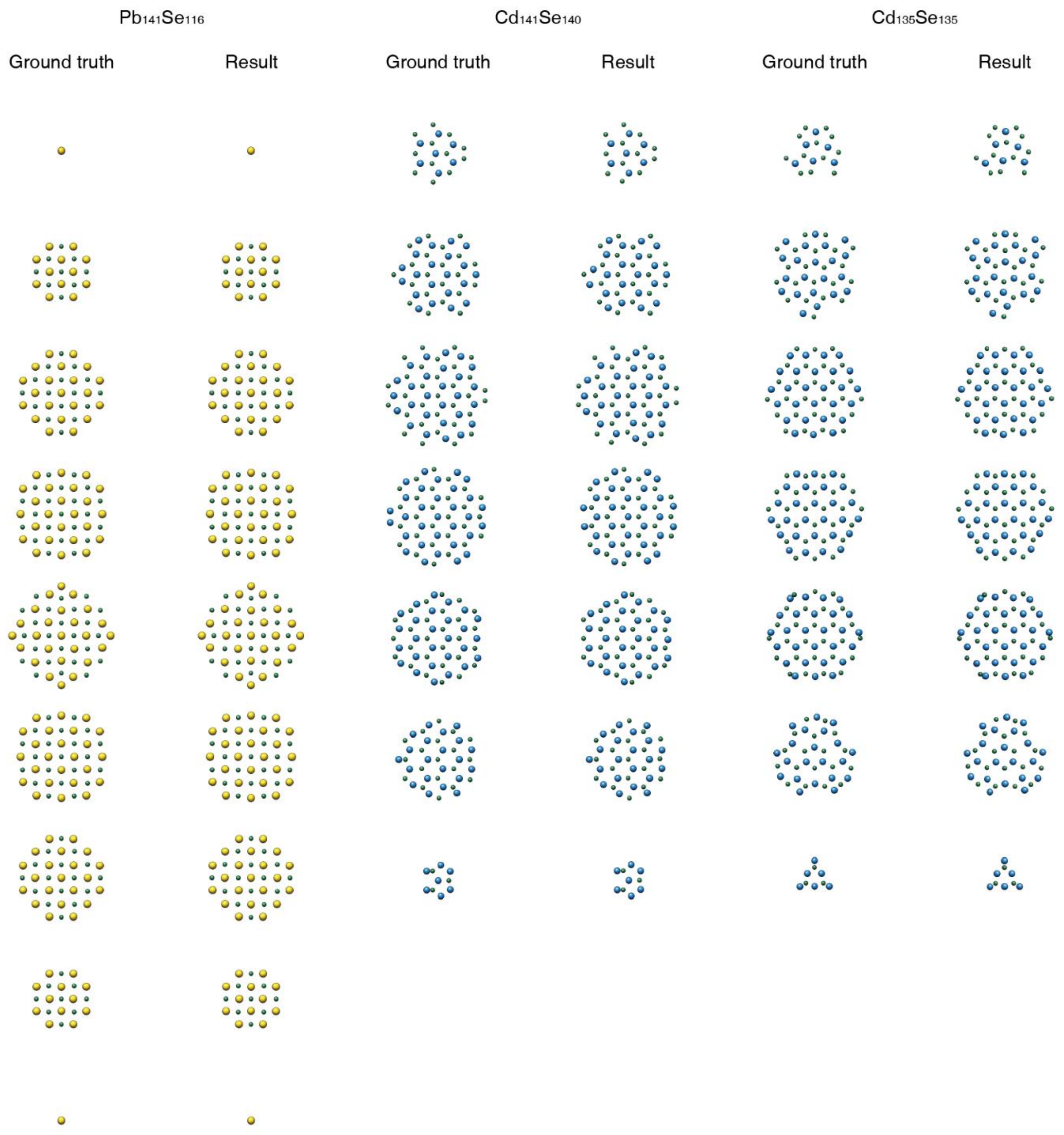

Figure S7. Slices of 3D atomic maps of ground truth and reconstructed atomic structures for PbSe and CdSe.

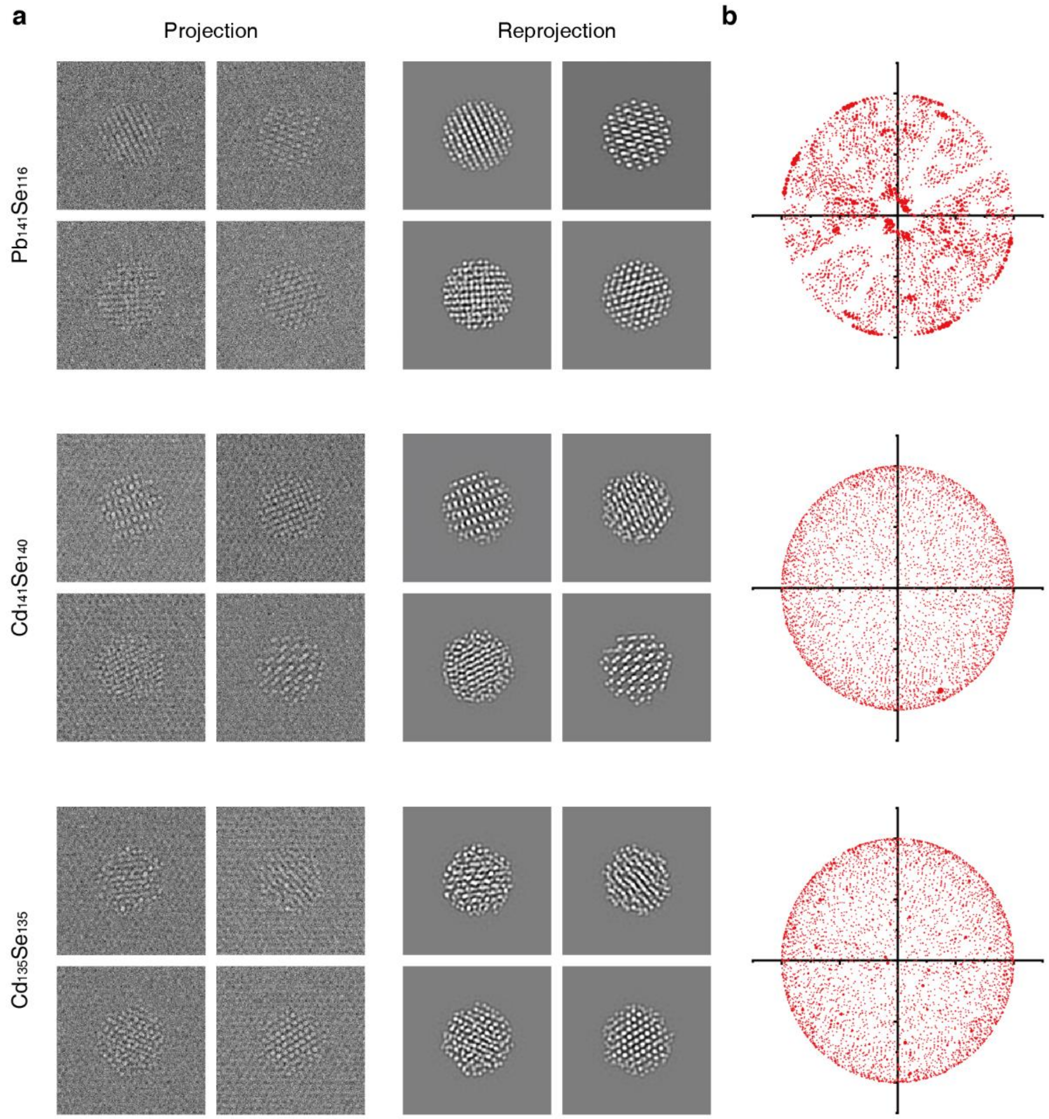

Figure S8. Validation of 3D reconstruction of ordered multi-element (PbSe and CdSe) nanoparticles. (a) Comparison between original projection images (simulated TEM images) and reprojected images of 3D Coulomb density maps. (b) Orientation coverage projected onto *xy*- planes.

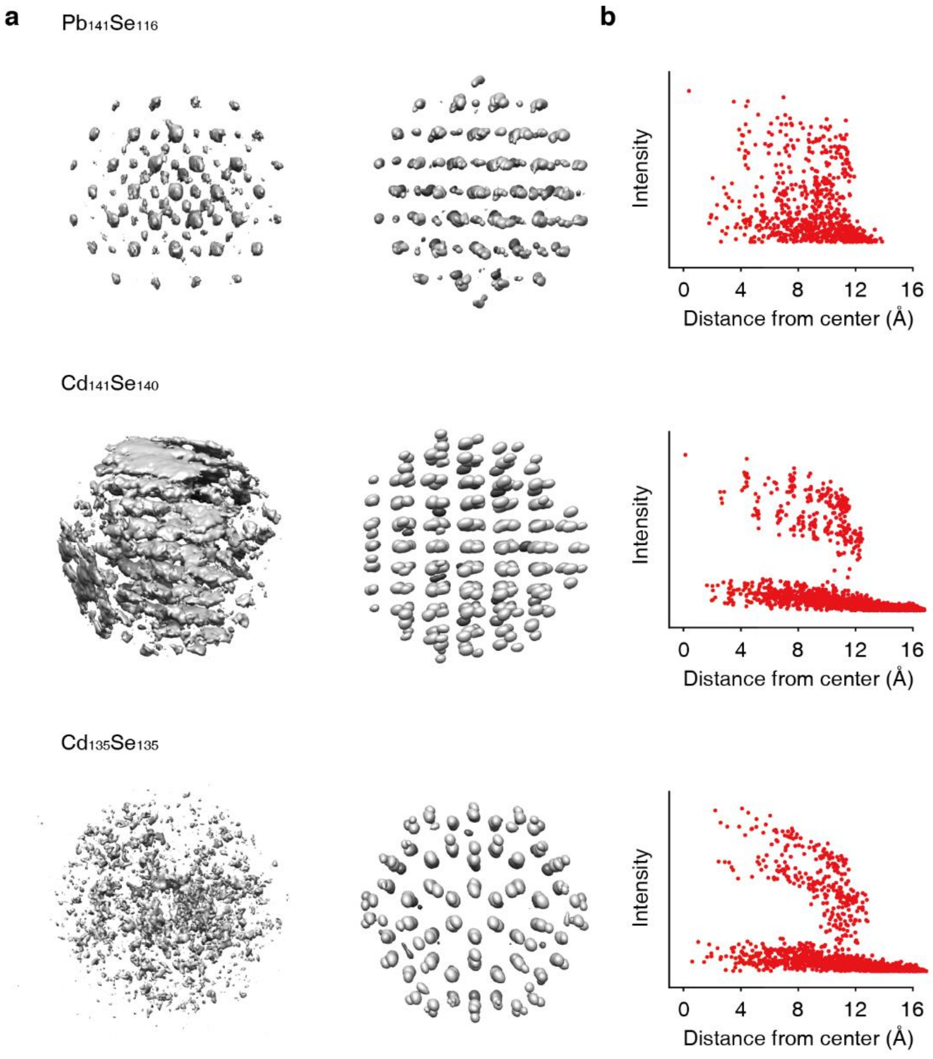

Figure S9. 3D reconstruction results using two-step reconstruction of ordered rocksalt $Pb_{141}Se_{116}$, wurtzite CdSe, and zinc blende CdSe. (a) 3D Coulomb density maps from each step. (b) The distance from the center of mass of the NP plotted as a function of local maximal intensity. The first volume map obtained by the two-step method had less lattice information than the single-step method and distinguishing between Pb(Cd) and Se was difficult.

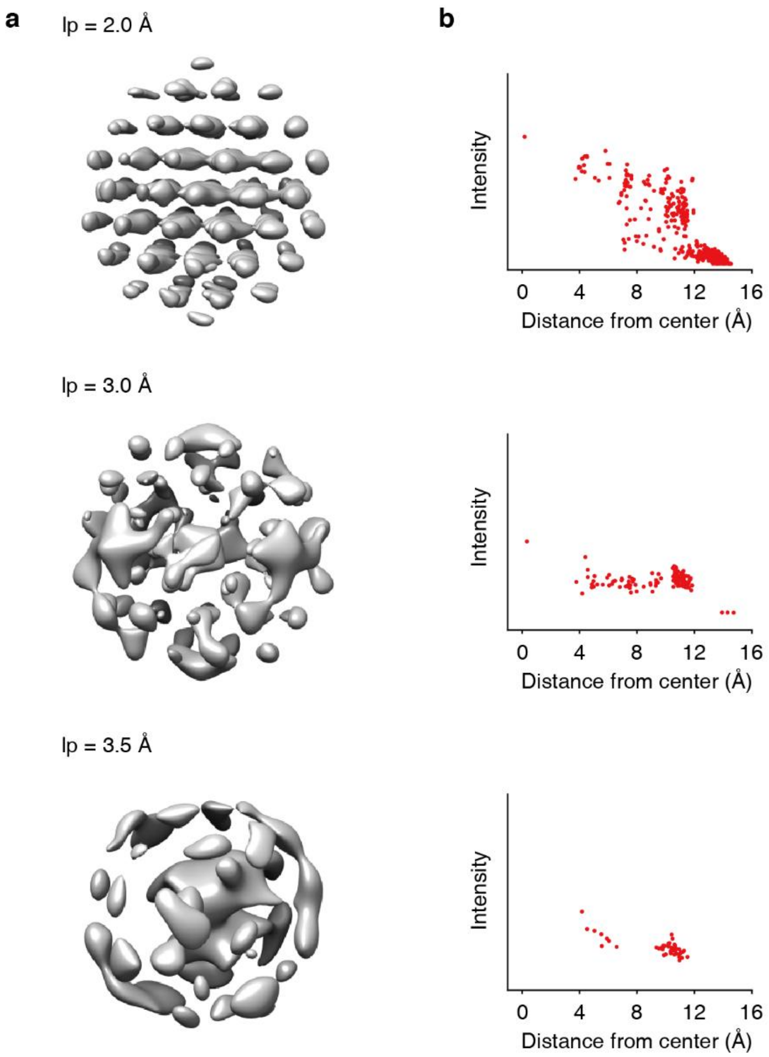

Figure S10. 3D reconstruction results for ordered rock-salt PbSe using different low-pass limits. (a) 3D Coulomb density maps. As the low-pass limit increases (more frequencies are omitted), the quality of the 3D reconstructions worsens. (b) The plot between distance from center of mass and local maxima intensity. As the low-pass limit increases, the number of elements and positions deviate more from the ground truth structure.

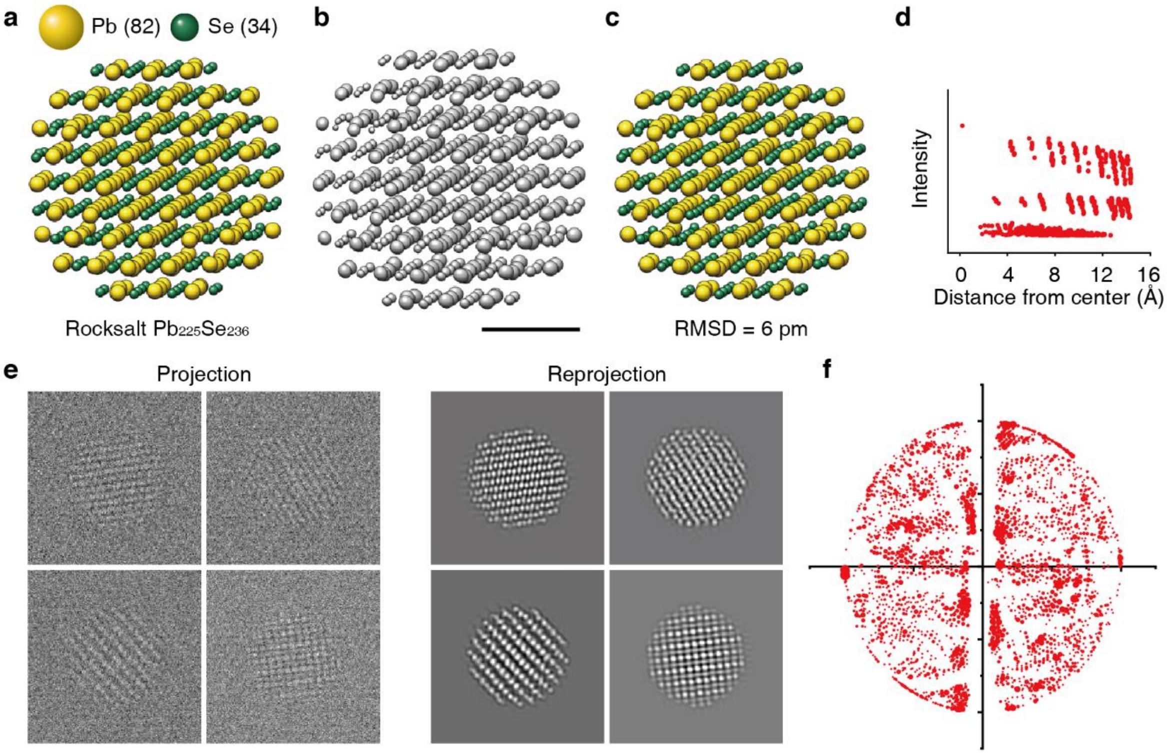

Figure S11. 3D reconstruction of 3 nm-sized ordered rocksalt PbSe nanoparticle. (a) The ground truth atomic structure. Yellow and green spheres correspond to Pb and Se atoms, respectively. (b) 3D Coulomb density maps obtained by single step reconstruction. Scale bar, 1 nm. (c) 3D atomic maps of reconstructed structures. Root mean square displacement (RMSD) between input and reconstructed structures are 6 pm. (d) Distance from the center of mass plotted as a function of local maximal intensity. (e) Comparison between original projection images (simulated TEM images) and reprojected images of 3D Coulomb density maps. (f) Orientation coverage projected onto *xy*- planes.

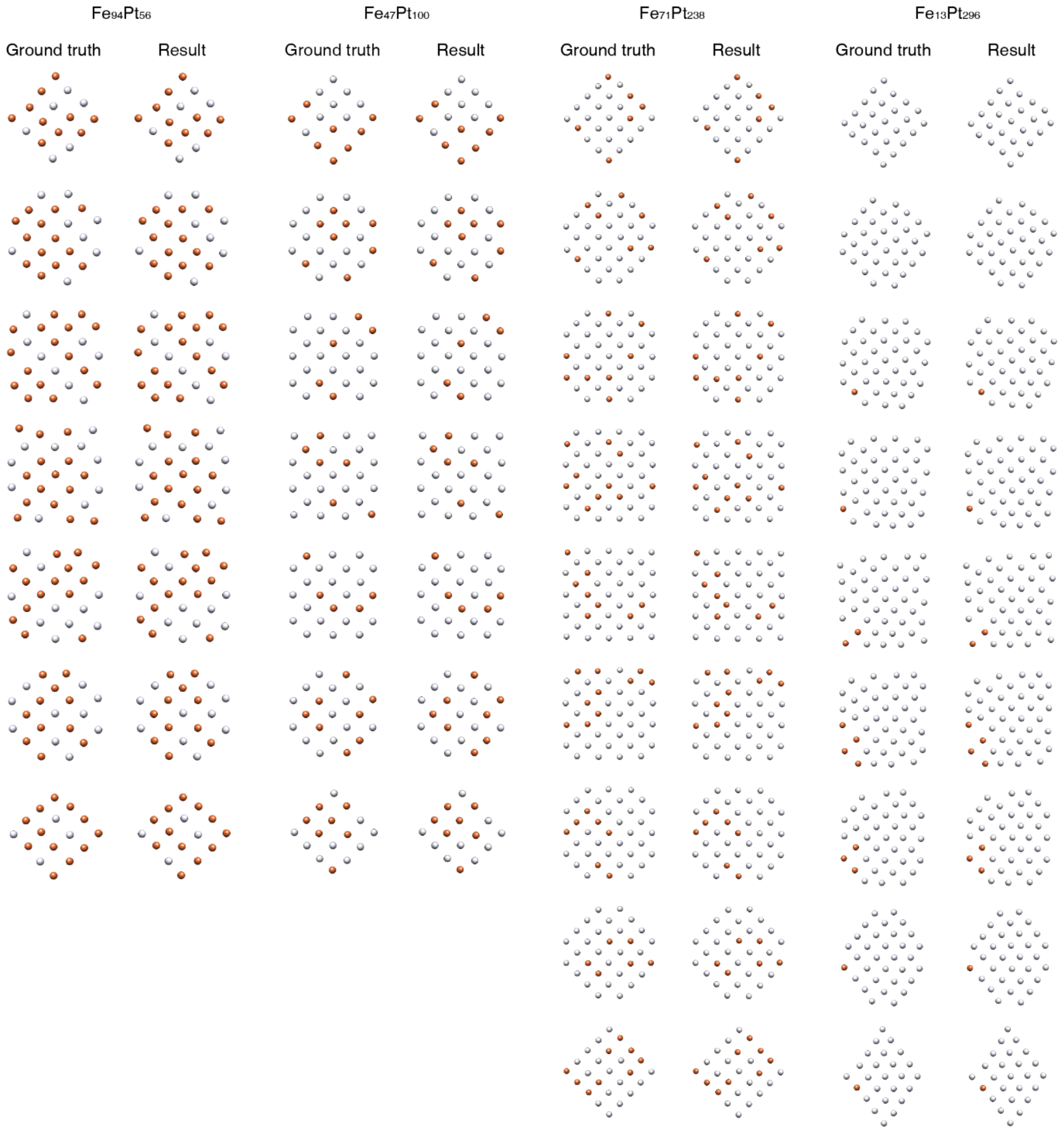

Figure S12. Slices of 3D atomic maps of ground truth and reconstructed atomic structures of four FePt nanoparticles.

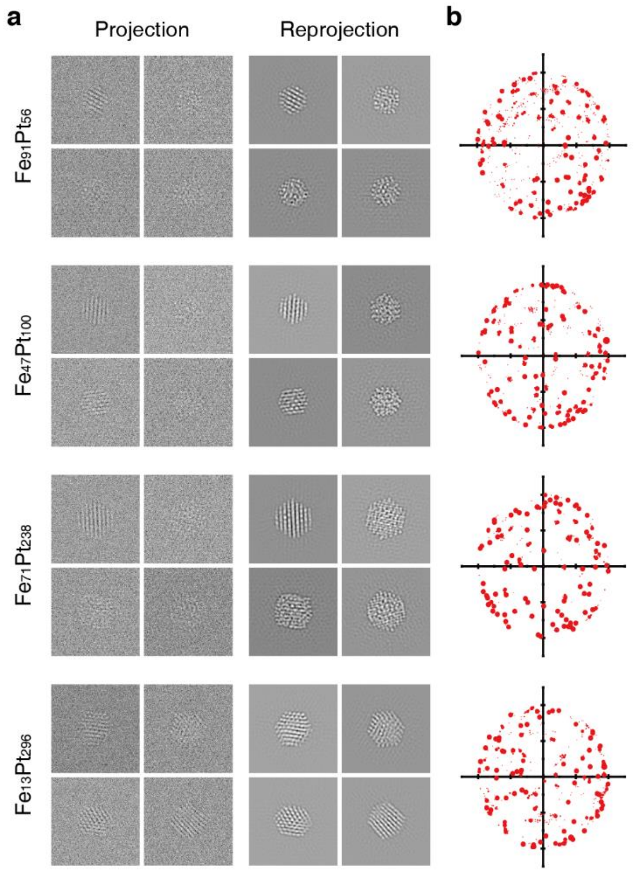

Figure S13. Validation of 3D reconstruction of four FePt nanoparticles. (a) Comparison between original projection images (simulated TEM images) and reprojections of the 3D Coulomb density maps. (b) Orientation coverage projected onto *xy*- planes.

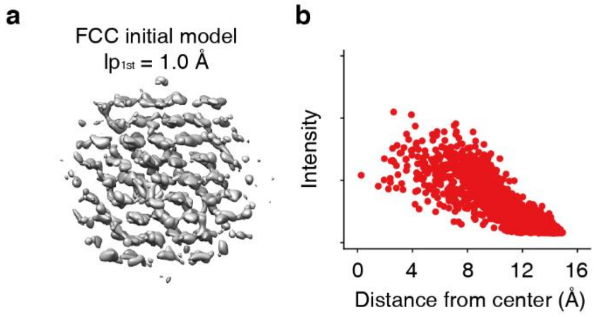

Figure S14. 3D reconstruction result using single-step reconstruction of disordered fcc $Fe_{71}Pt_{238}$. (a) 3D Coulomb density map. Used initial model and applied low-pass limit indicated. (b) The plot between distance from center of mass and local maxima intensity.

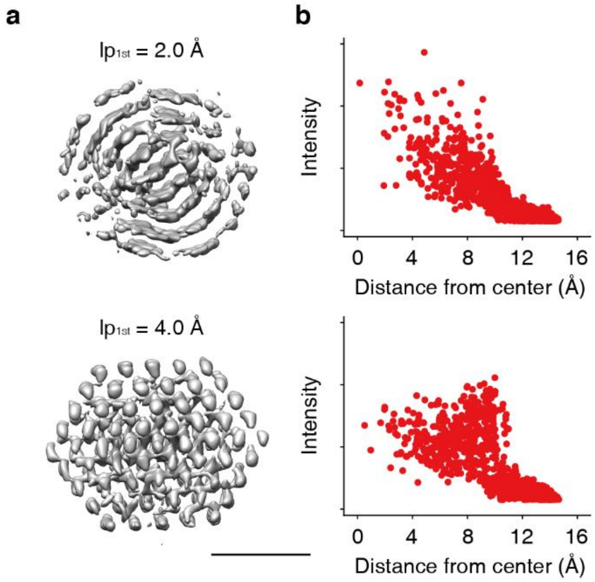

Figure S15. 3D reconstruction result of disordered fcc $Fe_{71}Pt_{238}$ with different low-pass filters. (a) 3D Coulomb density maps. Applied low-pass filter filters are described in angstrom unit. (b) Distance from the center of mass plotted as a function of local maximal intensity.

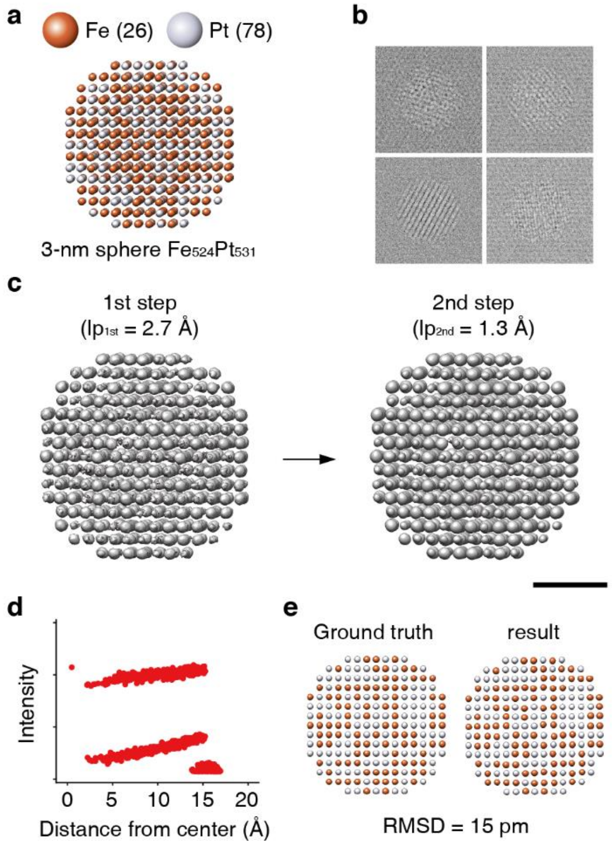

Figure S16. 3D reconstruction of 3 nm-sized disordered fcc FePt nanoparticle. (a) The ground truth atomic structure. Grey and orange spheres correspond to Pt and Fe atoms, respectively. (b) Representative simulated TEM images. (c) 3D Coulomb density maps obtained by two-step reconstruction. The applied low-pass filters for each step are depicted. (d) Distance from the center of mass plotted as a function of local maximal intensity. (e) A representative slice of 3D atomic maps of input structures and reconstructed structures. Root mean square displacement (RMSD) between input and reconstructed structures are 15 pm. Scale bar, 1 nm.

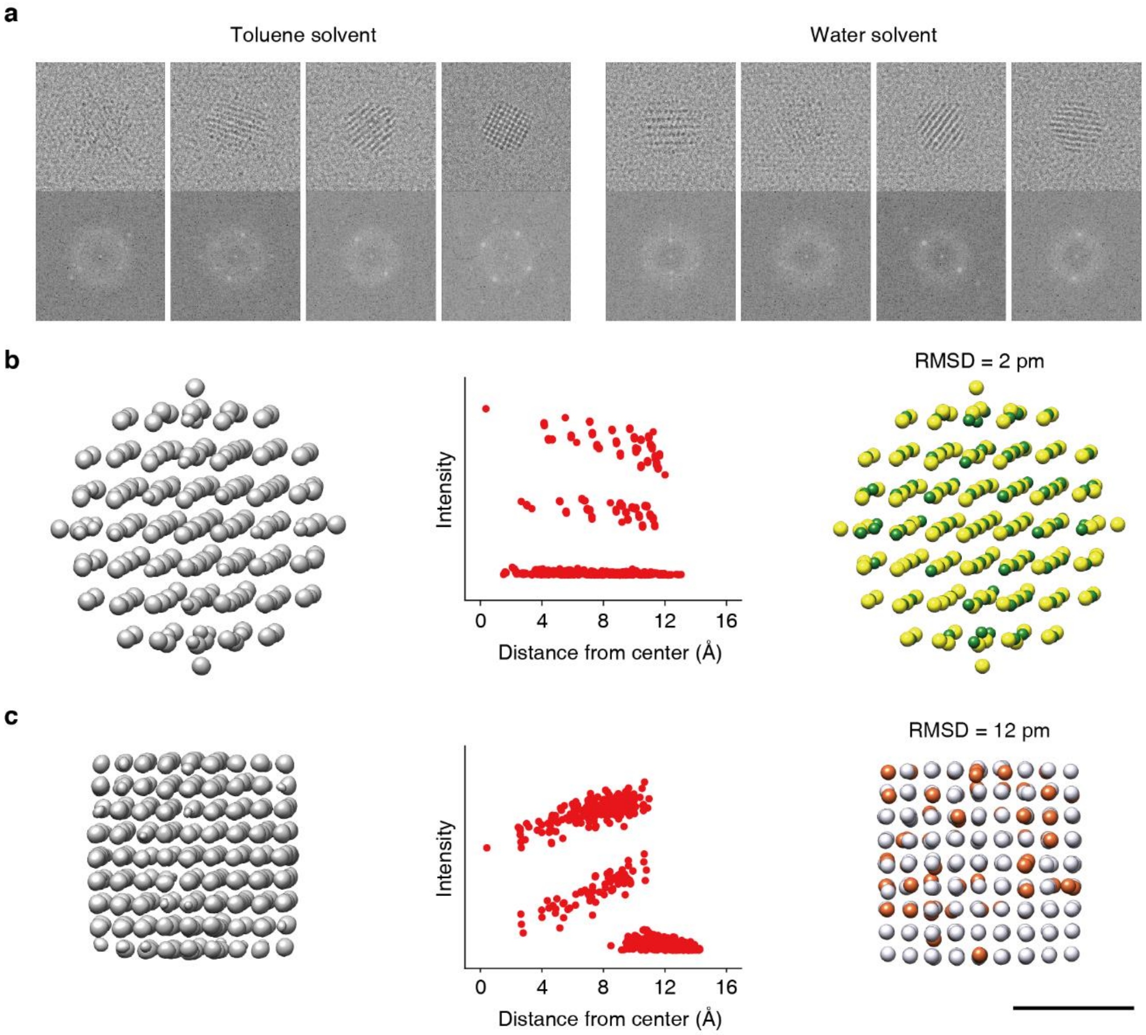

Figure S17. TEM simulation and 3D reconstruction results using different solvent. (a) Comparison between simulated TEM images, which sets solvent as toluene and water, respectively. 2D FFTs of the images are shown below the corresponding images. First two images for each set are from rocksalt PbSe nanoparticle and last two images are from disordered fcc FePt nanoparticle. (b and c) 3D reconstruction results of PbSe nanoparticle (b) and FePt nanoparticle (c) from simulated TEM images with water solvent. (right: 3D Coulombic density map, middle: intensity profile, left: atomic map) RMSD values are calculated between input and reconstructed structures. Scale bar, 1 nm.

References

45. Chen, M., Liu, J. P. & Sun, S. One-step synthesis of FePt nanoparticles with tunable size. *J Am Chem Soc* **126**, 8394–8395 (2004).

46. Wang, C., Daimon, H., Lee, Y., Kim, J. & Sun, S. Synthesis of monodisperse Pt nanocubes and their enhanced catalysis for oxygen reduction. *J Am Chem Soc* **129**, 6974–6975 (2007).

47. Dong, A. *et al.* A Generalized Ligand-Exchange Strategy Enabling Sequential Surface Functionalization of Colloidal Nanocrystals. *J Am Chem Soc* **133**, 998–1006 (2011).

48. Park, J. *et al.* Direct Observation of Wet Biological Samples by Graphene Liquid Cell Transmission Electron Microscopy. *Nano Lett* **15**, 4737–4744 (2015).
